# Severe Aortic Valve Stenosis and Pulmonary Hypertension: A Systematic Review of Non-Invasive Ways of Risk Stratification, Especially in Patients Undergoing Transcatheter Aortic Valve Replacement

**DOI:** 10.3390/jpm12040603

**Published:** 2022-04-08

**Authors:** Elke Boxhammer, Alexander E. Berezin, Vera Paar, Nina Bacher, Albert Topf, Sergii Pavlov, Uta C. Hoppe, Michael Lichtenauer

**Affiliations:** 1 Department of Internal Medicine II, Division of Cardiology, Paracelsus Medical University of Salzburg, 5020 Salzburg, Austria; e.boxhammer@salk.at (E.B.); v.paar@salk.at (V.P.); n.bacher@salk.at (N.B.); a.topf@salk.at (A.T.); u.hoppe@salk.at (U.C.H.); 2 Internal Medicine Department, State Medical University of Zaporozhye, 69035 Zaporozhye, Ukraine; aeberezin@gmail.com; 3 Department of Clinical Laboratory Diagnostics, State Medical University of Zaporozhye, 69035 Zaporozhye, Ukraine; escbm@escbm.org

**Keywords:** aortic valve stenosis, biomarker, cardiovascular imaging, echocardiography, pulmonary hypertension, sPAP, TAVR

## Abstract

Patients with severe aortic valve stenosis and concomitant pulmonary hypertension show a significantly reduced survival prognosis. Right heart catheterization as a preoperative diagnostic tool to determine pulmonary hypertension has been largely abandoned in recent years in favor of echocardiographic criteria. Clinically, determination of echocardiographically estimated systolic pulmonary artery pressure falls far short of invasive right heart catheterization data in terms of accuracy. The aim of the present systematic review was to highlight noninvasive possibilities for the detection of pulmonary hypertension in patients with severe aortic valve stenosis, with a special focus on cardiovascular biomarkers. A total of 525 publications regarding echocardiography, cardiovascular imaging and biomarkers related to severe aortic valve stenosis and pulmonary hypertension were analyzed in a systematic database analysis using PubMed Central^®^. Finally, 39 publications were included in the following review. It was shown that the current scientific data situation, especially regarding cardiovascular biomarkers as non-invasive diagnostic tools for the determination of pulmonary hypertension in severe aortic valve stenosis patients, is poor. Thus, there is a great scientific potential to combine different biomarkers (biomarker scores) in a non-invasive way to determine the presence or absence of PH.

## 1. Introduction

### 1.1. Prevalences

Severe aortic valve stenosis (AS) is the most common valvular heart disease requiring treatment in the elderly in the Western world [1]. In the course of demographic development and the aging population in developed countries, the number of cases will steadily increase. Among 75-year-olds, the prevalence of aortic valve stenosis is 6.6–18.2%, with echocardiographic evidence of severe AS in 1.1–5.7% [2]. The classic symptom triad consisting of angina, syncope and dyspnea indicates an advanced disease process. In particular, clinically new-onset dyspnea is indicative of increased blood backflow into the pulmonary circulation (consecutive backward failure), which sooner or later leads to successive changes in the vascular anatomy of the lung and thus to pulmonary hypertension (PH).

The simultaneous presence of both severe AS and PH is reported in the literature to be 48–75% [3] and is known to be associated with shortened long-term survival after surgical valve replacement (SVR) or transcatheter aortic valve replacement (TAVR) [4]. The gold standard for the detection of PH remains a right heart catheterization (RHC) examination with invasive hemodynamic measurements. For a long time, RHC, along with left heart catheterization, was an important part of the preoperative procedure regardless of whether SVR or TAVR was performed. Nowadays, this examination rarely finds its way into preoperative diagnostics in large cardiology centers and is reserved for selected special cases only. Therefore, noninvasive procedures such as echocardiography are needed to determine the presence or absence of PH.

### 1.2. Pathophysiology

The starting point for the pathophysiological mechanism of aortic valve stenosis is a significant reduction of the aortic valve opening area in most cases due to progressive calcification processes. From <1.0 cm^2^ on, severe AS is considered to exist. According to current European Society for Cardiology (ESC) guidelines, additional criteria are the mean pressure gradient across the valve (>40 mmHg) and the maximum velocity across the valve (>4.0 m/s) [5]. A consecutive increase in left ventricular pressure, which is necessary to eject blood to the periphery against the increased pressure gradient of the aortic valve, maintains cardiac output as well as systemic pressure. In this process, concentric hypertrophy of the left ventricular myocardium occurs primarily. This occurring hypertrophy leads to an increased rigidity of the left ventricle and thus to diastolic dysfunction. This in turn causes increased blood backflow from the left ventricle into the left atrium and further into the pulmonary circulation. The resulting pulmonary venous congestion leads to remodeling of the pulmonary vessels and consequently to an increase in pulmonary arterial pressure in the sense of PH [6,7].

The extent of aortic valve calcification, ventricular and pulmonary vascular remodeling and ultimately oxidative stress due to inflammatory processes can be detected, at least in part, at the molecular level by determining biomarkers [8]. In addition to known cardiovascular biomarkers such as Brain Natriuretic Peptide (BNP)/N-terminal prohormone of Brain Natriuretic Peptide (NT-proBNP) or troponin, markers of inflammation such as high sensitive CRP (hsCRP) as well as interleukin-8 (Il-8) and markers of oxidative stress such as the cytokine growth differentiation factor 15 (GDF-15) have shown important diagnostic value [9]. Especially in clinically asymptomatic patients, the determination of cardiovascular biomarkers, in addition to the gold standard of echocardiography, provides an additional diagnostic tool to assess severity and prognostic relevance. In addition to BNP and NT-proBNP, other molecular markers such as endothelin-1, vascular endothelial growth factor-D, and microRNAs play an important role in PH in the context of left heart disease (post-capillary PH) and thus also in patients with severe AS [10,11].

### 1.3. Definition of PH Using Invasive and Non-Invasive Techniques

The gold standard for the detection of PH is the performance of an RHC. The invasive determination of the important parameters such as mean pulmonary arterial pressure (mPAP), pulmonary artery wedge pressure (PAWP), diastolic pressure gradient (DPG) and pulmonary vascular resistance (PVR) allow a correct assignment into corresponding PH subtypes according to the currently valid ESC guidelines from 2015 [12]. PH can be excluded with an mPAP < 25 mmHg, whereas PH is present with an mPAP ≥ 25 mmHg. For further subdivisions into pre-capillary and post-capillary PH, the determination of PAWP plays a crucial role, with pre-capillary PH defined at a PAWP ≤ 15 mmHg and post-capillary PH at a PAWP > 15 mmHg. Occasionally, isolated studies [13] use LVEDP ≤ 15 mmHg vs. > 15 mmHg instead of PAWP as a distinguishing criterion. A further subdivision of post-capillary PH into isolated post-capillary (ipc-PH) and combined pre- and post-capillary PH (cpc-PH) is defined by the criteria of DPG and PVR. Either a PVR criterion ≤ 3 Wood units (WU) vs. > 3 WU or a DPG < 7 mmHg vs. ≥7 mmHg was used to differentiate between ipc-PH and cpc-PH. This classification reveals that patients with post-capillary PH and a PVR ≤ 3 WU + a DPG ≥ 7 mmHg or a PVR > 3 WU + a DPG < 7 mmHg cannot be classified as either ipc-PH or cpc-PH. To circumvent this discrepancy, isolated studies resorted to additional subgrouping of these patients [14].

At the Sixth World Symposium 2018 in Nice, a new PH definition was proposed, which has not found its way into the ESC guidelines yet [15]. In this definition, the mPAP threshold was decreased from ≥25 mmHg to >20 mmHg. However, PAWP of <15 mmHg vs. ≥15 mmHg remains as an unchanged criterion to distinguish between pre-capillary and post-capillary PH. Nevertheless, it is new that DPG is to be dropped as a criterion for the classification between ipc-PH and cpc-PH. By renewing the ESC guidelines, this important change should make unclassifiable post-capillary patients with a PVR ≤ 3 WU and a DPG > 7 mmHg or a PVR > 3 WU and a DPG ≤ 7 mmHg a thing of the past. Minor changes were also proposed for the ipc-PH as well as the cpc-PH classification, changing the PVR criterion to <3 WU instead of ≤3 WU and from >3 WU to ≥3 WU, respectively. The classification of pre-capillary PH should also be expanded to include the obligatory PVR criterion ≥ 3 WU in addition to PAWP ≤ 15 mmHg. For a better understanding, a direct comparison of the different PH classifications according to both ESC guidelines (2015) and Nice criteria (2018) is provided by means of a tabular presentation in Table 1.

However, the preoperative performance of RHC in patients with severe AS nowadays plays only a minor role in large, cardiological centers and is therefore no longer part of the preoperative standard. Therefore, echocardiography has to be given an important value regarding the noninvasive determination of PH. The basis of PH detection by echocardiography is the measurement of continuous wave Doppler over the tricuspid valve with analysis of the peak tricuspid regurgitation velocity (TRV). Taking into account the currently valid ESC guidelines, the presence of PH should be estimated on the basis of the TRV. TRV values ≤ 2.8 m/s are considered low risk and values of 2.9–3.4 m/s are considered intermediate risk for PH. Here, the guidelines recommend additional assessment of further echocardiographic “PH-signs” such as inferior vena cava (IVC) diameter, end-systolic right atrial area, early diastolic pulmonary regurgitation velocity or right ventricular outflow Doppler acceleration time. TRV values ≥ 3.5 m/s are associated with a very high risk of PH, so no further echocardiographic parameters need to be considered for risk assessment. In clinical practice, TRV is used together with right atrial pressure (RAP) to estimate systolic pulmonary arterial pressure (sPAP) by echocardiography. For this purpose, the simplified Bernoulli equation, sPAP = (4 × TRV^2^) + RAP, is used. RAP is estimated using the end-expiratory measured diameter of the IVC. With an IVC diameter ≥ 21 mm and a respiratory caliber fluctuation < 50%, a RAP of 15 mmHg (range: 10–20 mmHg) can be assumed. For an IVC diameter < 21 mm as well as a respiratory caliber fluctuation ≥ 50%, a RAP of 3 mmHg (range: 0–5 mmHg) is estimated. Other scenarios not corresponding to the above constellations are ascribed an intermediate value of 8 mmHg (range: 5–10 mmHg) [16,17,18].

Cardiovascular imaging is currently of minor importance, especially for the determination of post-capillary PH in left heart diseases. Cardiac CT is used to determine the main pulmonary artery (MPA) diameter with a cut-off value ≥ 29 mm, the ratio of MPA to ascending aorta (AA) named PA/AA_ratio_ with a cut-off value ≥ 1.0 and the ratio of segmental artery to segmental bronchus diameter, thus providing information about possible PH [19,20]. Cardiac MRI can be used to determine the size, structure, and function of the right ventricle and also to non-invasively assess the distensibility of pulmonary arteries [21].

### 1.4. Aim of the Review

The aim of the present review is to provide an overview of noninvasive options for the assessment of PH in patients with severe AS undergoing TAVR. In addition to echocardiography, other imaging modalities such as CT and MRI and, last but not least, cardiovascular biomarkers on a molecular level are analyzed.

## 2. Methods

A systematic database search was performed in PubMed Central^®^. Only English-language publications were included in this review. Search terms for the association between AS/PH and biomarkers, AS/PH and echocardiography as well as AS/PH and cardiovascular imaging are shown in Table 2.

**Figure 1:**
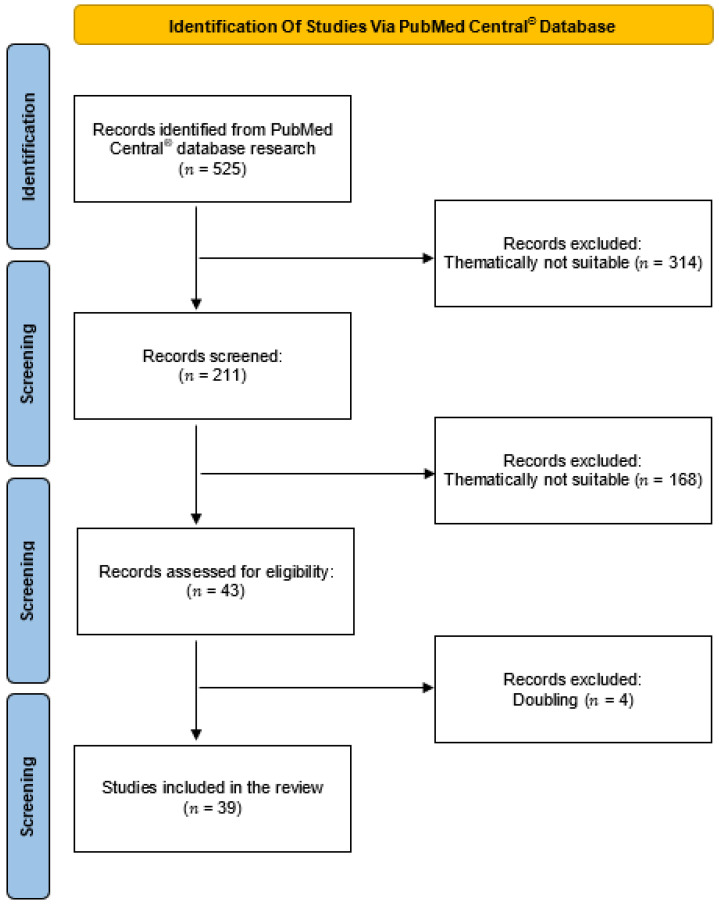
Flow diagram of the database search, screening, eligibility and inclusion of the studies (modified from the Preferred Reporting Items for Systematic Reviews and Meta-Analyses (PRISMA) guidelines.

To filter out appropriate studies for this review, the corresponding abstract was screened in addition to the title. Publications included were read in their entirety, whereas duplicate manuscripts were excluded. Reference lists of considered studies were also checked for further readings. This review was conducted based on the Preferred Reporting Items for Systematic Reviews and Meta-Analyses (PRISMA) guidelines (Figure 1) [22].

## 3. Results

### 3.1. Echocardiography

Echocardiography not only plays a crucial role in the detection of severe AS, but is also currently the method of choice in clinical practice to determine the presence or absence of PH in patients with severe AS. Some authors set the cut-off for PH at an sPAP ≥ 40 mmHg [23,24,25,26] and one study at an sPAP ≥ 42 mmHg [27], whereas other authors estimate the cut-off value slightly higher at ≥45 mmHg [28,29,30] or even ≥50 mmHg [31,32,33,34,35]. For example, Schewel et al. compared the echocardiographically obtained sPAP with invasively obtained sPAP using RHC in their study. The Pearson’s correlation coefficient of r = 0.820 was in a very satisfactory range. It was also shown in this study that a cut-off value ≥ 40 mmHg had better overall statistical quality criteria than a cut-off value ≥ 45 mmHg or ≥50 mmHg.

In some cases, the severity of PH was also attempted to be classified by the sPAP. In this regard, the common classification group I (no/mild PH): sPAP ≤ 40 mmHg, group II (moderate PH): sPAP 41–59 mmHg and group III (severe PH): sPAP ≥ 60 mmHg was applied [36,37]. Other studies, however, used TRV as the main criterion instead of sPAP, because the sometimes very individually determined estimation of RAP can be omitted. The typical classification group I (no/mild PH): TRV ≤ 2.8 m/s, group II (moderate PH): TRV 2.9–3.4 m/s and group III (severe PH): TRV ≥ 3.5 m/s was used in most of the studies analyzed here [38,39,40,41].

In most of the studies, the presence of PH was associated with a worse prognosis in terms of long-term survival. In particular, severe PH defined by sPAP ≥ 60 mmHg [42,43] or TRV ≥ 3.5 m/s [44] was an independent predictor of significantly faster patient demise in most studies. In a study that distinguished AS in terms of flow and gradient, the highest proportion of PH was seen in patients with high gradient [45].

The change in PH criteria, especially sPAP, before TAVR compared with after TAVR was also prognostic. In a large number of publications, any form of AVR resulted in a reduction in sPAP level and thus improved survival prognosis. Patients who showed persistently high sPAP levels after AVR or whose levels increased even further after AVR showed significantly increased 1-year and 2-year mortalities, respectively [46,47,48].

Special forms of echocardiography such as stress echocardiography or speckle tracking found their way into the literature only in one publication each in the context of severe AS and PH. Lancellotti et al. [49] showed in a collective of 105 patients, who underwent both resting echocardiography and stress echocardiography, that patients in stress echocardiography fulfilled the criteria for PH more frequently and were exposed to cardiac events of any kind significantly more often during the course of the study. When using speckle tracking echocardiography in patients with severe AS and PH, as Salas-Pacheco et al. [50] suggested, there is a possibility of increased occurrence of LA strain of the reservoir phase.

The publications with corresponding year of publication used regarding the context of severe AS, PH and echocardiography are shown in Table 3.

### 3.2. CT and MRI

#### 3.2.1. CT

As part of an adequate preoperative diagnosis before surgical or interventional aortic valve replacement in severe AS, CT angiography is performed to evaluate the aorta and other vessels near the heart. This CT imaging allows non-invasive conclusions about the presence of PH, for example, by assessing the MPA diameter or the PA/AA_ratio_. In their study, Eberhard et al. [51] examined 257 patients with severe AS undergoing TAVR who received RHC and divided the subjects into “no PH” and “PH” based on the detected mPAP. Subsequent measurements included MPA Diameter and PA/AA_ratio_, which revealed highly significant differences between the two groups. The combination of highest sensitivity and specificity was found with respect to MPA Diameter at values of 29–31 mm, which was very close to the cut-off value of ≥29 mm suggested by the ESC guideline. Chaturvedi et al. [52] demonstrated in their collective with severe AS patients a cut-off value of 30.5 mm with a sensitivity of 68.4% and a specificity of 82.7%. However, Eberhard et al. pointed out that although remodeling with consecutive enlargement of the pulmonary trunk occurs in severe AS due to chronic left heart strain, this parameter alone is not precise enough to detect non-invasive PH accurately. In addition, neither MPA Diameter nor PA/AAratio correlated significantly with patient outcome. This contrasted with the statement of Turner et al. [53], who found a relevant association with respect to 1-year survival, particularly in the complex calculation of MPA area. O’Sullivan et al. [54] used a similar patient population as Eberhard et al. (TAVR patients with RHC data as well as multi-detector computed tomography (MDCT) measurements). The results showed not only significantly higher MPA Diameter and PA/AA_ratios_ in PH patients, but also good correlation analyses with corresponding right heart catheterization data. As a major difference to the ESC guidelines, the optimal cut-off value for PA/AA_ratio_ was set at 0.80 (sensitivity 56.0%, specificity 88.0%). A relevant new approach was recently published by Sudo et al. [55] who related the MPA diameter to the body surface area (PA/BSA) and thus defined a good predictor for PH detection. The extent to which the distensibility of the pulmonary artery, which can be determined in CT diagnostics, has diagnostic value for the assessment of PH [56] remains to be clarified in further studies.

The publications with corresponding year of publication used regarding the context of severe AS, PH and CT are shown in Table 4.

#### 3.2.2. MRI

Cardiac MRI (cMRI) does not play a relevant role in preoperative diagnosis before either SVR or TAVR. Therefore, few studies with a small number of subjects focused on the detection of post-capillary PH in the setting of severe AS using MRI imaging. In 2019, Gumauskiene et al. [57] investigated the impact of severe AS with additional PH on left ventricular (LV) parameters in particular. Of 30 patients, 23% showed severe AS and PH, with significantly higher LV end-diastolic volume index, larger LV fibrosis area and lower LV global longitudinal strain on cMRI. In particular, LV fibrosis area and LV global longitudinal strain were valuable predictors for detecting the presence of PH in severe AS. In another study in 2021, the same working group led by Gumauskiene et al. [58] showed in a very similar patient population with concurrent endomyocardial biopsy that histologically detectable diffuse myocardial fibrosis correlated positively with LV dilatation and negatively with LV dysfunction, global longitudinal strain and circumferential strain, respectively.

The publications with corresponding year of publication used regarding severe AS, PH and MRI are shown in Table 4.

### 3.3. Biomarkers

Patients undergoing SVR or TAVR may present with several cardiovascular risk factors that can affect clinical outcome after the procedure [59]. In addition, co-existing adverse cardiac remodeling, PH and heart failure (HF) continue to have a strong impact on the clinical status, quality of life and survival of patients after successful TAVR [60,61]. Therefore, new-onset atrial fibrillation, TIA/stroke, myocardial infarction, acute kidney injury, severe bleeding and advanced HF were found to be strong predictors of poor clinical outcomes and higher rates of re-admission after TAVR even in AS patients with moderate risks [62,63]. Overall, the impact of numerous cardiovascular factors, age and gender, comorbidities, post-TAVR complications (contrast-induced nephropathy, bleeding) and procedure-related factors (permanent pacemaker implantation) on all-cause and cardiovascular mortality appears to be quite complex. It is pointless to reduce the impact on prognosis after TAVR to a single factor, even if it is as valuable as HF, atrial fibrillation or PH. However, it is noteworthy that age-related conditions (hypertension, atherosclerosis), gender and a profile of other cardiovascular risk factors provide the background for progression and re-occurrence of HF and PH [64,65].

It should mean that adverse cardiac remodeling associated with moderate to severe AS plays a central role in the development of other cardiovascular and cerebrovascular events, such as atrial fibrillation, TIA/stroke, acute coronary syndrome/acute myocardial infarction, conversion of HF with preserved ejection fraction to HF with reduced ejection fraction and fatal arrhythmias/sudden cardiac death [66,67]. PH is not only frequently accompanied by adverse cardiac remodeling, but it is also promoted by cumulative effects of HF, atrial fibrillation and other factors such as preload and afterload, skeletal muscle weakness and metabolic disease (diabetes, obesity and thyroid dysfunction) [68,69,70,71,72]. In this context, cardiac biomarkers reflecting biomechanical stress (natriuretic peptides (NPs), myocardial damage (high-sensitivity cardiac troponins), inflammation (soluble suppression of tumorigenicity 2 (sST2), fibrosis (galectin-3, GDF-15), oxidative stress and endothelial dysfunction are considered useful for clinicians to improve risk stratification models to better manage their patients.

NPs are functional antagonists of the renin–angiotensin–aldosterone system and provide adaptive effects on water and sodium homeostasis, blood pressure, vascular integrity, diuresis and renal function [73]. In clinical conditions associated with increased cardiac stretching, NPs have been measured in elevated concentrations. Nowadays, NPs are powerful predictors of all-cause and cardiovascular mortality, urgent hospitalization and readmission due to progression of HF, and they are also established diagnostic biomarkers of HF in clinical routine [74,75]. Elevations of NPs in circulation is common for both AS and PH [76,77,78,79]. Previously, it has been found that elevated plasma levels of BNP (>475 pg/mL) before and after TAVR were the strongest independent predictor of all-cause and cardiovascular mortality [80].

Furthermore, in surviving patients after TAVR, plasma BNP levels were found to decrease 30 days after TAVR, and a delay was associated with premature death in patients [81]. Therefore, a trend toward a decline in BNP levels after TAVR is thought to provide additional prognostic information for patients. Mizutani et al. [82] confirmed this assumption and found that elevated BNP levels at discharge were not only associated with 2-year mortality after TAVR, but also inclusion in a multiple predictive score along with other clinical variables sufficiently improved the predictive accuracy for 2-year mortality. A recent systematic review and meta-analysis by White et al. [83] found that elevated BNP levels compared with lower baseline biomarker levels were predictors of all-cause mortality in patients with severe AS. Of these biomarkers, elevated BNP, NT-proBNP, high-sensitive cardiac troponin T (hs-cTnT) and galectin-3 levels before TAVR were positively associated with increased all-cause mortality in an overall population of patients with AS. Another meta-analysis of currently available clinical trials showed that high baseline levels of NT-proBNP predicted increased mid-term mortality but not early mortality in patients with aortic stenosis after TAVR [84].

Elevated levels of hs-cTnT have provided solid evidence of their prognostic capabilities in patients with various cardiovascular diseases, including those with moderate-to-severe AS, independent of HF and PH [85]. Although elevated circulating hs-cTnT levels (>10 ng/L) in patients with severe aortic stenosis were strongly associated with high risks of cardiovascular events within one year [86,87], a multiple biomarker model constructed from NPs and hs-cTnT is considered more predictive for these patients [88]. This approach seems promising to guide the treatment of AS, including TAVR [89]. For example, Chorianopoulos et al. [90] reported that pre- and post-interventional hs-cTnT levels positively correlated with 1-year mortality rates in patients with severe AS, independent of successful aortic valve replacement, while there are numerous controversial data from the clinical setting reflecting the fact that only pre-TAVR hs-cTnT levels predicted all-cause death in these patients [91,92]. One-year hs-cTnT ≥ 39.4 pg/mL and NT-proBNP levels > 300 pg/mL, along with other factors such as male sex, eGFR < 60 mL/min/1.73 m^2^, and chronic obstructive pulmonary disease, were identified as independent predictors of long-term mortality in TAVR patients [93].

A meta-analysis of 19 clinical trials (a total of 7555 patients undergoing TAVR) examining the effects of pre- and postprocedural hs-cTnT levels on mortality rates provided evidence that high pre-TAVR levels were significantly associated with an increase in both short-term (30-day) and intermediate-term mortality, whereas no association was found between high post-procedural hs-cTnT levels and 30-day mortality [94]. However, a strong positive association was found between high post-TAVR hs-cTnT levels and an increase in midterm mortality. As a strong predictor of all-cause mortality and cardiovascular mortality in patients with cardiovascular disease complicated with PH [95], hs-TnT levels not only show strong correlations with hemodynamics [96], but also appear to be a promising indicator of events after TAVR [97,98]. However, this evidence requires further investigation in large clinical trials [99].

Galectin-3 reflects the intensity of myocardial fibrosis and cardiac biomechanical stress, microvascular inflammation, oxidative stress and vascular osteogenesis in atherosclerosis [100,101,102]. Additionally, it is involved in the pathogenesis of AS and is considered a predictive biomarker as well as a molecular target for therapies in patients with severe AS [103]. Elevated galectin-3 levels are strongly positively related to severity of adverse cardiac remodeling, LV hypertrophy, dynamic changes in LV geometry [104,105] and global LV longitudinal strain [106]. In a small clinical trial, elevated galectin-3 levels before TAVR showed a tendency to predict all-cause mortality in patients with severe AS [107]. Importantly, galectin-3 levels were not related to clinical status, other biochemical parameters or cardiac hemodynamic characteristics, including LV ejection fraction and LV mass index. In another clinical study, circulating galectin-3 levels were shown to correlate well with sPAP and PAWP as well as all-cause mortality and cardiovascular events one year after TAVR. In addition, AS patients with a galectin-3 level of >17.8 ng/mL had a higher risk of death [108]. Finally, the authors concluded that the addition of galectin-3 to NT-proBNP provides additive predictive value for risk stratification. In patients with severe AS undergoing TAVR, elevated galectin-3 levels ≥ 8.71 ng/mL predicted adverse clinical outcomes (all-cause mortality or readmission for worsening HF) only when carbohydrate antigen 125 (CA-125) was additionally elevated [109]. Thus, the authors found a potential molecular interaction between galectin-3 and CA-125, the cause of which remains to be elucidated in detail.

Giritharan et al. [110] examined a profile of serum biomarkers BNP, galectin-3, GDF-15, sST2, osteoprotegerin, microRNA-19b and microRNA-21 in patients undergoing TAVR and found that this signature provided a more accurate risk assessment than echocardiographic parameters. Zhang et al. [111] reported the results of a systematic review and meta-analysis in which they found that circulating galectin-3 levels before TAVR predicted an increased risk of all-cause mortality. It is possible that galectin-3, which shows promising and robust results in AS patients at high surgical risk, is a practically useful biomarker for predicting short- and long-term clinical outcomes after valve replacement [112].

Markers of collagen metabolism such as circulating N-terminal propeptide of procollagen I (PINP), C-terminal telopeptide of collagen I (CITP), N-terminal propeptide of procollagen III (PIIINP), microRNA-19b and microRNA-21 have been extensively studied as predictive biomarkers in patients with AS over the past decade [113]. Although CITP and PIIINP were found to be strongly associated with HF, especially HFrEF and cardiac dysfunction, circulating collagen metabolites were not accurate surrogate biomarkers for myocardial fibrosis in patients with AS [114]. However, the concentration of circulating PIIINP correlated positively with PAWP and inversely with LV ejection fraction and stroke volume index [115]. At the same time, downregulated expression of microRNA-19b, which elucidates collagen fibril cross-linking, predicted altered myocardial collagen network in AS patients, especially in those who had HF [116]. MicroRNA-21, which is a regulator of fibrosis and reflects an association with pressure overload in aortic stenosis patients, may be a promising biomarker for myocardial fibrosis [117]. Overall, these biomarkers are still under investigation, and their role in predicting events after TAVR, including HF, progression of PH, and atrial fibrillation, remains uncertain [118], whereas there is evidence that levels of another microRNA-133a, reflecting turnover of myocardial collagen metabolism, before TAVR was able to predict regression of LV hypertrophy after TAVR [119].

A member of the interleukin (IL)-1 receptor family, sST2, is considered a potent modulator of hypertrophic, inflammatory and fibrotic myocardial responses as well as aortic and aortic valve calcification [120,121,122]. Elevated sST2 levels are an established biomarker for predicting outcomes in HF [123,124]. Lancellotti et al. [125] reported that peak sST2 is an independent predictor of cardiovascular events in patients with AS. Fabiani et al. [126] found that sST2 ≥ 284 ng/mL had the best accuracy for predicting altered global longitudinal strain in patients with severe AS. However, sST2 levels before TAVR were not significantly different between HF patients and AS patients with normal EF (EF ≥ 50%) [127]. Therefore, there were no correlations between sST2 levels and NT-proBNP concentration and parameters of AS severity [128]. Patients with severe AS who had poor clinical outcome after TAVR had significantly higher sST2 levels before TAVR and higher NT-proBNP levels before and 6 months after TAVR [129]. Finally, pre-TAVR sST2 levels were found to be strong predictors of postprocedural cardiovascular events and 1-year mortality in these patients [130,131,132]. Indeed, the addition of soluble urokinase plasminogen activator receptor (suPAR) to sST2 significantly improved the predictive power of each biomarker for cardiovascular outcomes after TAVR [133]. Using a prospective registry of patients with aortic stenosis, Lindman et al. [134] showed that a multiple biomarker model constructed from sST2 together with BNP and galectin-3 was more predictive of 1-year and 2-year mortality rates in patients undergoing TAVR than either biomarker alone. Thus, sST2 levels before TAVR could serve as a specific and sensitive predictive biomarker for AS patients.

GDF-15 is a multifunctional cytokine that belongs to the TGF-beta superfamily and is involved in senescence and modulation of adverse cardiac remodeling, myocardial fibrosis and endothelial dysfunction by suppressing the inflammatory response and potentiating tissue repair [135,136]. GDF-15 levels correlated with indices of LV dysfunction, including reduced global longitudinal strain, left ventricular mass and lower Katz score [137]. Previously, predictive ability for cardiovascular events, all-cause mortality and cardiovascular mortality was demonstrated in HF patients [138]. GDF-15 levels were found to be sufficiently elevated in patients with mild to severe AS compared to patients without this disease [139]. Moreover, a strong association of GDF-15 levels with the degree of aortic stenosis was found [140]. Kim et al. [141] reported that elevated GDF-15 levels were associated with maladaptive cardiac remodeling and increased mortality after TAVR. Moreover, GDF-15 levels were superior to NT-proBNP in TAVR risk stratification and better than other biomarkers, such as galectin-4, von Willebrand factor, interleukin-17 receptor A, transferrin receptor protein 1 and pro-protein convertase subtilisin/kexin type 9, in predicting postoperative outcome [142,143].

The publications with corresponding year of publication used regarding the context severe AS, PH and biomarkers are shown in Table 5.

## 4. Discussion and Conclusions

As can be seen from the results section and especially from the number of included publications, echocardiography in particular is considered to be of greatest value for the non-invasive assessment of PH in patients with severe AS. With the estimation of sPAP, an approbate tool is available to determine, among other things, the severity of PH, although this is also dependent to some extent on the experience of the examiner and the ultrasound quality of the patient.

While cardiac MRI for the assessment of PH generally has only experimental approaches, almost all patients receive imaging by CT before surgical or interventional aortic valve replacement. Here, MPA diameter and PAA/AA_ratio_ have emerged as potential PH parameters.

Although there is a large amount of scientific data on cardiovascular biomarkers and severe AS, few papers can be found that additionally highlight biomarker expression from the perspective of post-capillary PH in the setting of AS. Gumauskiene et al. [144], Maeder et al. [145] and Calin et al. [146] described significantly increased BNP and NT-proBNP levels, respectively, in patients with severe AS and PH compared with patients in whom no PH could be detected by echocardiography or RHC. Gumauskiene et al. also described this relationship for GDF-15 and saw a moderate correlation (r = 0.508; *p* = 0.003) between GDF-15 and echocardiographically determined sPAP. Combining NT-proBNP and GDF-15 raised the positive correlation with sPAP to r = 0.640.

In summary, the data base on severe AS, concomitant PH and biomarker levels is modest. Therefore, large-scale clinical trials need to investigate the following:

- Which biomarkers have the potential to provide information about the presence of PH in patients with severe AS?
- What cut-off values for the detection of PH do these biomarkers have?
- Should biomarker scores be developed and not only solitary biomarkers be determined in order to detect PH in a non-invasive way?
- How do plasma concentrations of biomarkers change after surgical or interventional valve replacement in patients with additional PH and does this have relevant implications for survival prognosis?

## Figures and Tables

**Table 1:** Determination of PH according to current ESC Guidelines (2015) and according to the Sixth World Symposium on Pulmonary Hypertension (2018). Mean pulmonary arterial pressure, (mPAP); Diastolic pressure gradient (DPG); Pulmonary vascular resistance (PVR); Pulmonary capillary wedge pressure (PCWP).

**Determination Of PH According to Current ESC Guidelines (2015)**	
**PH Subtypes**	**Hemodynamics**
pre-capillary PH	mPAP ≥ 25 mmHg PCWP ≤ 15 mmHg
isolated post-capillary PH	mPAP ≥ 25 mmHg PCWP > 15 mmHg PVR ≤ 3 WU DPG < 7 mmHg
combined pre- and post-capillary PH	mPAP ≥ 25 mmHg PCWP > 15 mmHg PVR > 3 WU DPG ≥ 7 mmHg
**Determination Of PH According To 6th World Symposium On Pulmonary Hypertension (2018)**	
**PH Subtypes**	**Hemodynamics**
pre-capillary PH	mPAP > 20 mmHg PCWP ≤ 15 mmHg PVR ≥ 3 WU
isolated post-capillary PH	mPAP > 20 mmHg PCWP > 15 mmHg PVR < 3 WU
combined pre- and post-capillary PH	mPAP > 20 mmHg PCWP > 15 mmHg PVR ≥ 3 WU

**Table 2:** Search terms used for provided review.

Search Terms	Search Results	Included Results
**Echocardiography**		
1. aortic stenosis AND pulmonary hypertension AND echocardiography	385	28
**Cardiovascular Imaging**		
1. aortic stenosis AND pulmonary hypertension AND computed tomography	57	6
2. aortic stenosis AND pulmonary hypertension AND mri	46	2
**Biomarkers**		
1. aortic stenosis AND pulmonary hypertension AND biomarkers	21	1
2. aortic stenosis AND pulmonary hypertension AND BNP	7	2
3. aortic stenosis AND pulmonary hypertension AND sST2	0	0
4. aortic stenosis AND pulmonary hypertension AND suPAR	0	0
5. aortic stenosis AND pulmonary hypertension AND gdf-15	1	0
6. aortic stenosis AND pulmonary hypertension AND gdf-11	0	0
7. aortic stenosis AND pulmonary hypertension AND galectin-3	0	0
8. aortic stenosis AND pulmonary hypertension AND microrna	1	0
9. aortic stenosis AND pulmonary hypertension AND h-fabp	0	0
10. aortic stenosis AND pulmonary hypertension AND troponin	7	0
11. aortic stenosis AND pulmonary hypertension AND ca-125	0	0

**Table 3:** Included studies evaluating the context of severe AS, PH and echocardiography.

Echocardiography				
Authors	Year	N	Population	Findings
Malouf et al. [44]	2002	47	Resting Echocardiography Severe AS Severe PH was defined when TRV ≥ 4.0 m/s in echocardiography	Severe PH was an independent predictor of perioperative mortality
Kapoor et al. [42]	2007	626	Resting Echocardiography Severe AS Severe PH was defined when sPAP ≥ 60 mmHg in echocardiography	Patients with sPAP ≥ 60 mmHg had a significantly smaller aortic valve area, a significantly lower LVEF and a significantly higher mitral E/A velocity ratio
Pai et al. [43]	2007	119	Resting Echocardiography Severe AS referred to AVR Severe PH was defined when sPAP ≥ 60 mmHg in echocardiography	AVR in patients with severe AS and PH led to a relevant survival benefit
Saraiva et al. [23]	2010	70	Resting Echocardiography Severe AS PH was defined when sPAP ≥ 40 mmHg in echocardiography	Patients with severe AS and PH presented with greater LV diameters, E/A ratio, E-wave velocity, LV mass index, reversed atrial wave velocity and LA volume 1 month after AVR LA function improved significantly
Lancellotti et al. [49]	2012	105	Resting and Stress Echocardiography Severe AS PH was defined when sPAP > 50 mmHg in resting echocardiography PH was defined when sPAP > 60 mmHg in stress echocardiography	PH in stress echocardiography was significantly more frequent than in resting echocardiography Presence of PH in stress echocardiography was associated with reduced cardiac event-free survival Presence of PH in stress echocardiography was an independent predictor of cardiac events
Mutlak et al. [31]	2012	216	Resting Echocardiography Severe AS PH was defined when sPAP ≥ 50 mmHg in echocardiography	Presence of PH led to a reduced LVEF and an impaired LV diastolic function Mortality in patients with PH was significantly higher
Luçon et al. [36]	2014	2435	Resting Echocardiography Severe AS referred to TAVR 3 Groups: sPAP < 40 mmHg; sPAP 40–59 mmHg; sPAP ≥ 60 mmHg in echocardiography	1-year mortality was higher in group II and group III compared to group I sPAP ≥ 40 mmHg was identified as an independent predictor of all-cause mortality
Medvedofsky et al. [32]	2014	122	Resting Echocardiography Severe AS referred to TAVR PH was defined when sPAP ≥ 50 mmHg in echocardiography	Patients with severe AS and PH had smaller aortic valve areas, greater degrees of mitral or tricuspid regurgitation and lower LVEF TAVR led to a reduction of sPAP level COPD was an independent predictor of post TAVR PH Presence of PH post TAVR was associated with a significantly higher 2-year mortality
Ahn et al. [24]	2014	189	Resting Echocardiography Moderate and Severe AS PH was defined when sPAP ≥ 40 mmHg	Patients with PH had a higher prevalence of diabetes, a lower LVEF, a larger LA volume and a smaller aortic valve area PH complicated AS independently by systolic and diastolic dysfunction
Barasch et al. [27]	2014	550	Resting Echocardiography Severe AS PH was defined when sPAP ≥ 42 mmHg	Mild to moderate pulmonary hypertension was an independent risk factor in patients undergoing AVR
Durmaz et al. [37]	2014	70	Resting Echocardiography Severe AS referred to TAVR 3 Groups: sPAP < 40 mmHg; sPAP 40–59 mmHg; sPAP ≥ 60 mmHg in echocardiography	After TAVR sPAP of group II and III decreased significantly TAVR led to a significant and permanent decrease of in sPAP
Bishu et al. [46]	2014	277	Resting Echocardiography Severe AS referred to TAVR Tertiles: sPAP ≤ 35 mmHg; sPAP 36–48 mmHg; sPAP; sPAP ≥ 49 mmHg	Patients in group III had worst diastolic dysfunction and more often chronic lung diseases Being in group III was an independent risk factor of long-term mortality
Barbash et al. [33]	2015	415	Resting Echocardiography Severe AS referred to TAVR 2 Groups: No/mild PH—sPAP ≤ 50 mmHg; moderate/severe PH—sPAP > 50 mmHg in echocardiography	Patients with moderate/severe PH had more often mitral valve regurgitation and right heart failure Patients with moderate/severe PH had higher 30-day and higher 1-year mortality sPAP was an independent predictor of 1-year mortality
D’Ascenzo et al. [25]	2015	674	Resting Echocardiography Severe AS referred to TAVR PH was defined when sPAP ≥ 40 mmHg in echocardiography	sPAP ≥ 40 mmHg was associated with a higher 30-day mortality Improvement of sPAP post TAVR was associated with a better overall outcome
Mascherbauer et al. [34]	2015	465	Resting Echocardiography Severe AS referred to AVR PH was defined when sPAP > 50 mmHg in echocardiography	Patients with tricuspid regurgitation had a significant higher probability of PH
Salas-Pacheco et al. [50]	2016	72	Speckle-tracking echocardiography 42 patients with moderate and severe AS PH was defined when sPAP > 40 mmHg in echocardiography	Strain of reservoir phase was mainly associated with PH Each decrease in one unit of strain of reservoir phase increased 6% the PH probability
Nijenhuis et al. [38]	2016	591	Resting Echocardiography Severe AS referred to TAVR 3 Groups: TRV ≤ 2.8 m/s; TRV 2.9–3.4 m/s; TRV ≥ 3.5 m/s in echocardiography	Group III was an independent predictor of 30-day mortality and 2-years morality
Hernandez-Suarez et al. [28]	2017	30	Resting Echocardiography Severe AS referred to TAVR PH was defined when sPAP ≥ 45 mmHg in echoccardiography	LV mass index and LA volume index were significantly elevated in patients with severe AS and PH Longitudinal measures of RV systolic function (TAPSE ans systolic velocity) were clearly reduced
Kleczysnki et al. [39]	2017	148	Resting Echocardiography Severe AS referred to TAVR 3 Groups: TRV ≤ 2.8 m/s; TRV 2.9–3.4 m/s; TRV ≥ 3.5 m/s in echocardiography	Group III presented with higher NYHA classifications levels and had more frequently a history of previous stroke Presence of PH (TRV ≥ 3.5 m/s) was not identified as an independent predictor of all-cause mortality at follow-up
Levy et al. [40]	2017	1019	Resting Echocardiography Severe AS referred to AVR 3 Groups: TRV ≤ 2.8 m/s; TRV 2.9–3.4 m/s; TRV ≥ 3.5 m/s in echocardiography	Group 3 (TRV ≥ 3.5 m/s) exhibited excess mortality in comparison to Group 1 (TRV ≤ 2.8 m/s) or Group 2 (TRV 2.9–3.4 m/s)
Masri et al. [29]	2018	407	Resting Echocardiography and RHC Severe AS referred to TAVR PH pre TAVR was defined when mPAP ≥ 25 mmHg in RHC PH post TAVR was defined when sPAP ≥ 45 mmHg in echocardiography	Patients with persistent presence of PH 1 month post TAVR had a significantly higher 2-year mortality
Kandels et al. [45]	2018	306	Resting Echocardiography Severe AS referred to AVR 4 Groups: Low-flow, low gradient AS; normal-flow, low gradient AS; low-flow, high gradient AS, normal-flow, high gradient AS PH was defined when sPAP > 35 mmHg in echocardiography	PH was significantly more often present in patients with high gradient AS
Rozenbaum et al. [35]	2019	97	Resting Echocardiography Severe AS referred to TAVR PH was defined when sPAP ≥ 50 mmHg	Patients with severe AS and PH were presented with higher PVR (echocardiographically determined) PVR ≥ 2.5 WU was an independent predictor of all-cause mortality
Schewel et al. [26]	2020	1400	Resting Echocardiography and RHC Severe AS PH was defined when sPAP ≥ 40 mmHg in echocardiography PH was defined when mPAP ≥ 25 mmHg in RHC	sPAP of RHC and echocardiography correlated well (r = 0.820) Bland Altman analysis showed a measurement accuracy of 80.6%
Ujihira et al. [47]	2020	242	Resting Echocardiography Severe AS referred to TAVR PH post TAVR was divided in 3 groups: Initial sPAP > +5 mmHg; initial sPAP ±5 mmHg; initial sPAP < −5 mmHg	Group I showed significantly higher mortality than group II or III Hospitalization rate after TAVR was significantly higher in group I than group II or III
Strachinaru et al. [48]	2020	170	Resting Echocardiography Severe AS referred to TAVR PH was defined when TRV ≥ 2.9 m/s in echocardiography	TAVR procedure led to a significantly decrease in TRV and thus to a lower PH detection
Cladellas et al. [41]	2020	429	Resting Echocardiography Severe AS referred to AVR 3 Groups: TRV ≤ 2.8 m/s; TRV 2.9–3.4 m/s; TRV ≥ 3.5 m/s in echocardiography	TRV ≥ 3.5 m/s was an independent predictor of all-cause mortality
Weber et al. [30]	2021	205	Resting Echocardiography and RHC Severe AS referred to AVR PH pre AVR was defined when mPAP ≥ 25 mmHg in RHC PH post AVR was defined when sPAP > 45 mmHg in echocardiography	TAVR reduced presence of PH 15 months post TAVR Patients with persistent presence of PH post TAVR had higher mPAP, PCWP and PVR in pre TAVR RHC

**Table 4:** Included studies evaluating the context of severe AS, PH and cardiovascular imaging.

CT and MRI				
Authors	Year	N	Population	Findings
Eberhard et al. [51]	2017	257	CT and RHC Severe AS referred to TAVR 161 patients with PH via RHC (mPAP ≥ 25 mmHg)	MPA diameter was significantly enlarged in patients with severe AS and PH Anterior pericardial recess was significantly enlarged in patients with severe AS and PH Pleural effusion was a predictor of higher 2-year mortality
O’Sullivan et al. [54]	2018	139	CT and RHC Severe AS referred to TAVR 114 patients with PH via RHC (mPAP ≥ 25 mmHg)	PA/AA_ratio_ correlated well with mPAP and sPAP PA/AA_ratio_ is a moderate predictor of PH detection Optimal cut-off of PA/AA_ratio_ was 0.80
Gumauskiene et al. [57]	2019	30	MRI and Echocardiography Severe AS 7 patients with PH via echocardiography (sPAP ≥ 45 mmHg)	Patients with PH had a higher LV end diastolic volume index, a larger LV fibrosis area and a lower LV global longitudinal strain
Colin et al. [56]	2020	100	CT and RHC 31 patients with severe AS PH via RHC (mPAP ≥ 25 mmHg)	Distensibility of pulmonary artery was lower in patients with PH Distensibility of pulmonary artery correlated negatively with mPAP
Turner et al. [53]	2021	402	CT and Echocardiography Severe AS referred to TAVR PH via echocardiography (sPAP ≥ 50 mmHg)	MPA area was associated with higher 1-year mortality Cut-off value for MPA area as a predictor of 1-year mortality was ≥ 7.40 cm^2^
Chaturvedi et al. [52]	2021	165	CT and RHC Severe AS referred to TAVR 85 patients with PH via RHC (mPAP ≥ 25 mmHg)	MPA diameter was higher in patients with PH Cut-off value of MPA diameter detecting PH was 30.5 mm
Gumauskiene et al. [58]	2021	34	MRI, Echocardiography and Endomyocardial Biopsy Severe AS referred to AVR 9 patients with PH via echocardiography (sPAP ≥ 45 mmHg)	Higher extent of myocardial fibrosis was detected in PH patients Myocardial fibrosis correlated with LV dilatation, LV dysfunction, global logitudinal and circumferential strain
Sudo et al. [55]	2022	770	CT Severe AS referred to TAVR	PA/BSA was a good predictor of PH detection Large PA/BSA value was associated with higher 2-year mortality

**Table 5:** Included studies evaluating the context of severe AS, PH and biomarkers.

Biomarkers				
Authors	Year	N	Population	Findings
Gumauskiene et al. [144]	2018	60	NT-proBNP, GDF-15 Severe AS referred to SVR 13 patients with PH via echocardiography (sPAP ≥ 45 mmHg)	NT-proBNP ≥ 4060 ng/L was associated with elevated sPAP GDF-15 ≥ 3393 pg/mL was associated with elevated sPAP
Maeder et al. [145]	2018	252	BNP Severe AS referred to AVR 111 patients with PH via RHC (mPAP ≥ 25 mmHg)	Higher BNP levels were associated with higher mPAP and PVR A higher BNP level is a possible predictor of the presence of combined pre- and post-capillary pulmonary hypertension
Calin et al. [146]	2020	108	BNP (available in 45 patients) Severe AS referred to AVR 20 patients with PH via echocardiography (sPAP ≥ 40 mmHg)	Patients with severe AS and PH had significantly higher BNP values

## Data Availability

The data presented in this study are available on request from the corresponding author.
